# 

**Published:** 1902-01-18

**Authors:** 


NOTICE TO CORRESPONDENTS.

All MSS., Letters, Books for Review, and other matters intended for the Editor should be addressed THE EDITOR, "THE HOSPITAL," THE HOSPITAL BUILDING, 28 & 29 SOUTHAMPTON STREET, STRAND, W.C.

The Editor cannot undertake to return rejected MS., even when accompanied by stamped directed envelope.

**Notice to Advertisers and Subscribers.**

All Advertisements, Orders for Copies of THE HOSPITAL, and Business Communications should be addressed to **THE MANAGER (Not to the Editor),** THE HOSPITAL, 28 & 29 Southampton Street, Strand, London, W.C.

The Cost of Subscription, post free, is as follows:—Per week, 2½d.; per quarter, 2s. 8d.; per half-year, 5s. 3d.; per annum, 10s. 6d. Foreign:—Per annum, 15s. 2d., payable, in all cases, in advance.

**NOTICE.—Vol. XXX. of "The Hospital" (April 1901, to September 1901), in handsome cloth binding can now be obtained at the office of this Journal, price, 7s. 6d. Cases for binding the Numbers contained in this Volume 1s. 6d. Index to Vol. XXX., 2½d., post free.**

**The Monthly Part for January is now ready. Price 6d., by post 8d.**

### BOOKS RECEIVED.

Adam and Charles Black.

"The Englishwoman's Year-book and Directory, 1902." Edited by Emily Janes.

The Scientific Press.

"Poison Romance and Poison Mystery." New Edition. By C. J. S. Thompson, F.R.Hist.S.

### THE STUDENT OF MEDICINE.

### APPOINTMENTS.

**Daniel Henry Beegling,** B.A.Sydney, M.B., C.M.Edin. appointed Surgeon to the London and North-Western Railway Company for Huddersfield and District. **J. T. Bell,** M.B., B.S.Dur., appointed Medical Officer of the Fifth District of the Wycombe Union. **F. E. Bissell,** L.R.C.P., L.R.C.S.Edin. appointed District Medical Officer of the Dore Union. **J. G. Boon,** L.R.C.P., L.R.C.S.I., appointed Certifying Factory Surgeon for the Borough of Wenlock, County Salop. **Richard V. Brews,** L.R.C.P., L.R.C.S.I., appointed District Medical Officer of the Woolwich Union. **A. Tennyson Brown,** M.B., C.M.Aberd., appointed Medical Officer of Health to the Horbury Urban District Council. **Frederick W. Collingwood,** L.R.C.P.Lond., M.R.C.S., appointed Anæsthetist to the Samaritan Free Hospital. **Harold Collinson,** M.B., M.R.C.S., L.R.C.P., appointed Resident Surgical Officer to the General Infirmary at Leeds. **Harrison Cripps,** F.R.C.S.Eng., appointed Surgeon to St. Bartholomew's Hospital. **Wm. H. Cuthbert,** L.R.C.P., L.R.C.S.Edin., appointed Medical Officer of Health for the Frinton-on-Sea Urban District. **T. B. Dakin,** M.R.C.S., L.R.C.P.Lond., appointed Certifying Factory Surgeon for the Ulceby District of Lincolnshire. **Stanley Edward Denyer,** C.M.G., M.A., M.D., B.C.Cantab., appointed Assistant Demonstrator of Anatomy in the University of Cambridge (Lent Term). **W. A. Eden,** M.R.C.S., L.R.C.P.Lond., appointed Medical Officer of the Eighth District of the Bodmin Union. **Arthur B. Harris,** M.B., Ch.B.Oxon., appointed Medical Officer of Health for the Loughton Urban District. **Wheelton Hind,** M.D.Lond., F.R.C.S., appointed Honorary Senior Surgeon to the North Staffordshire Infirmary and Eye Hospital, Stoke-on-Trent. **P. Kinmont,** M.B., F.R.C.S.E., appointed Senior House-Surgeon to the County Hospital, Lincoln. **Frank Burnand Mudd,** M.R.C.S., L.R.C.P., appointed Honorary Anæsthetist to the Throat Hospital, Golden Square, **W. J. E. O'Connor,** M.B., Ch.B., R.U.I., appointed Medical Officer of Health for the combined Districts of Leicestershire, Rutland, and Warwick. **Edmund Owen,** F.R.C.S., appointed Consulting Surgeon and Surgeon-in-Chief to the French Hospital and Dispenary. **John Pardoe,** M.B., M.R.C.S., appointed Surgical Assistant to St. Peter's Hospital, Covent Garden. **William Robb,** M.B., C.M.Glasg., appointed Medical Officer of Health for the Irthlingborough Urban District. **Francis C. Rogers,** F.R.C.S., L.R.C.P.Edin., appointed Medical Officer of Health for the Erith Urban District. **W. D. Spanton,** F.R.C.S., appointed Consulting Surgeon to the North Staffordshire Infirmary and Eye Hospital, Stoke-on-Trent.

